# Ignition and Flame Stabilization of a Strut-Jet RBCC Combustor with Small Rocket Exhaust

**DOI:** 10.1155/2014/675498

**Published:** 2014-01-20

**Authors:** Jichao Hu, Juntao Chang, Wen Bao

**Affiliations:** Harbin Institute of Technology, Xidazhi Street No. 92, Harbin, Heilongjiang 150001, China

## Abstract

A Rocket Based Combined Cycle combustor model is tested at a ground direct connected rig to investigate the flame holding characteristics with a small rocket exhaust using liquid kerosene. The total temperature and the Mach number of the vitiated air flow, at exit of the nozzle are 1505 K and 2.6, respectively. The rocket base is embedded in a fuel injecting strut and mounted in the center of the combustor. The wall of the combustor is flush, without any reward step or cavity, so the strut-jet is used to make sure of the flame stabilization of the second combustion. Mass flow rate of the kerosene and oxygen injected into the rocket is set to be a small value, below 10% of the total fuel when the equivalence ratio of the second combustion is 1. The experiment has generated two different kinds of rocket exhaust: fuel rich and pure oxygen. Experiment result has shown that, with a relative small total mass flow rate of the rocket, the fuel rich rocket plume is not suitable for ignition and flame stabilization, while an oxygen plume condition is suitable. Then the paper conducts a series of experiments to investigate the combustion characteristics under this oxygen pilot method and found that the flame stabilization characteristics are different at different combustion modes.

## 1. Introduction

For potential economic benefit, reusable single-stage-to-orbit aerospace vehicle has been a research focus over several decades. In order to maintain optimal propulsion efficiency within a wide flight regime, several kinds of combined-circle engines have been studied [1–3]. The most promising strategy is known as the Rocket Based Combined Circle engine (RBCC). By embedding a rocket engine in a supersonic combustion ramjet (scramjet) combustor, four different modes can be operated in a single flow path: (1) ejector-rocket-mode for take-off and initial acceleration, (2) ramjet-mode for supersonic accelerating flight, (3) scramjet-mode for hypersonic accelerating flight, and (4) rocket-mode for further acceleration and space flight [4–6]. As different operating modes are coupled in a single flow path, the combustion chamber must make sure that the combustion is stable and reliable during the mode transition.

The rocket has played important multiple roles during the whole flight range. In the take-off period, the rocket is the main source of engine thrust, most of the fuel reacted in the rocket chamber with the taken oxygen, due to the fuel rich stated of the rocket exhaust, there will be some un-reacted fuel left. This part of the fuel will continue to react with the breathed incoming air flow and we call it the second combustion. Flame stabilization of the second combustion at this ejector mode is mainly accomplished by the large mount of hot rocket gas with much high chemical activity [7, 8]. As the flight Mach number continues to rise, the engine enters into ramjet mode, the total mass flow rate of the rocket begins to decrease, and the thrust tends to be generated by the second combustion. Some research work has been done to investigate thrust optimization law by changing the fuel proportion injected into the rocket chamber and into the second air flow [9] and the result has demonstrated this trend. However the decrease of the rocket exhaust mass flow rate may affect the flame stabilization of the second combustion.

In these researches, rocket plume is fuel rich and the fuel used in these works is hydrogen. The high activity of the hydrogen makes sure that the flame stabilization is not a serious problem. Problem is getting much more complex when the fuel is liquid kerosene. Compared with hydrogen used in many researches, kerosene has some advantage for its high energy density and easy to handle, but its additional evaporating process and the relative low chemical activity will need higher combustion organization technical requirements [10–13]. This should be paid attention by the researchers when designing a hydrocarbon fuel RBCC combustor.

This paper tries to investigate the influence of the rocket base on the second reacting flow using liquid kerosene experimentally with a relative small total mass flow rate of the rocket exhaust. For this purpose, a strut-jet model is built by embedding a rocket with a rectangle chamber in a fuel injection strut and mounted in the center of a flush wall supersonic combustor. This preliminary foundation experimental work is to investigate the influence of the different rocket plume characteristics (fuel rich or oxygen rich) on the flame stabilization of the second combustion.

## 2. Facility Description

**Figure 1:**
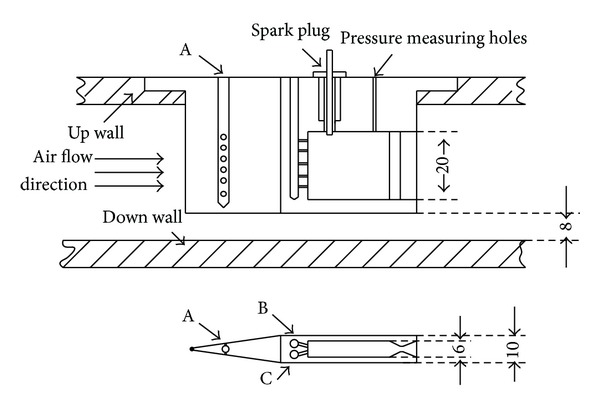
Structure of the strut-jet unit: mm.

Structure of the strut-jet is shown in Figure 1 and can be divided by two sections: The first part is a wedge, the angel of the wedge is 20 degrees. There are a set of orifices drilled in the first part for injection of the kerosene to the RBCC combustor, this is, marked as second kerosene (K_2_ for short). The second part is the rocket base next to the wedge. The cross-section of the rocket chamber is rectangle with the area is 6 × 20 mm^2^ and the length is 30 mm. Throat area of the laver nozzle is 3 × 20 mm^2^ and the exit area is 6 × 20 mm^2^. Kerosene for the rocket chamber is injected from orifice B which is marked as first kerosene (K_1_ for short) and oxygen for the rocket is injected from orifice C. The rocket is lit by a spark plug.

**Figure 2:**
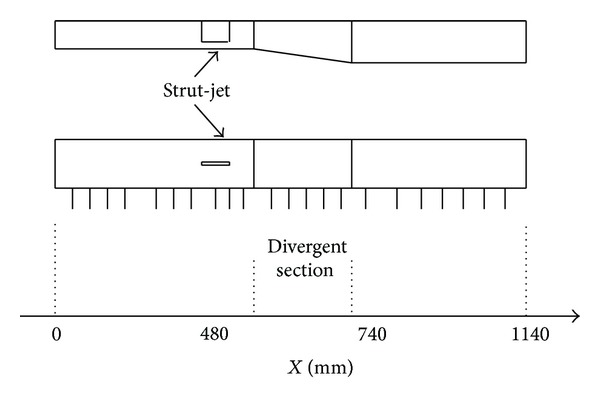
Structure of the strut-jet RBCC combustor model unit: mm.

The strut-jet is mounted in a supersonic combustor and the combustor is heat-sink combustor model made of stainless steel and can be divided into four parts: the first part is 480 mm long with a constant 50 × 100 mm^2^ rectangular cross-section, and this part is used to simulate the isolator. A 260 mm long expansion part is connected with the isolator, expanding from 50 mm × 100 mm to 70 mm × 100 mm. The third part is a 400 mm long constant area section. The whole length of the combustor is 1140 mm. The structure of the RBCC combustor model is shown in Figure 2.

There are 22 pressure transducers mounted on the combustor side wall to reflect the performance of the second combustion. The range of the pressure transducers is 0-1 MPa and the max error is ±0.25% in full scale and the frequency of the transducers is 0.5 kHz. There is also a pressure transducer located in the chamber of the rocket to measure the chamber pressure of the rocket, which is a main parameter to reflect the rocket plume characteristic.

## 3. Results and Discussion

### 3.1. Ignition with Fuel Rich and Oxygen Rich Plume

**Figure 3:**
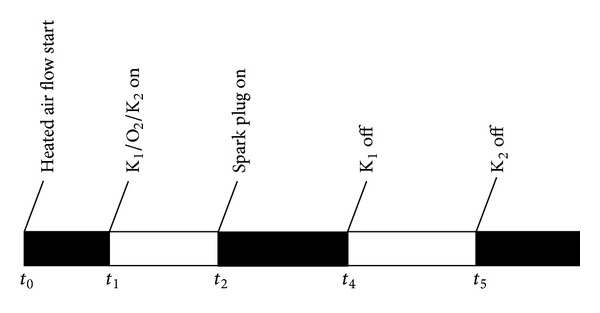
Time sequence of the experiment.

In this section, two kinds of rocket plume are generated. One plume is with 15 g/s kerosene and 15 g/s oxygen; this mass flow rate ratio is used in reference [9] and in this condition, this plume is fuel rich. The other plume is pure oxygen with a mass flow rate of 15 g/s. The experiment time sequence is shown in Figure 3. After the heated air flow is founded, three solenoid valves are turned on: the kerosene injected into the strut-jet (K_1_), the oxygen injected into the strut-jet, and the kerosene injected into the main combustor for second combustion (K_2_); the mass flow rate of K_2_ is corresponding to an ER of 0.3. This ER refers to the equivalence of the RBCC combustor. At *t*_2_, the spark plug in the strut-jet is turned on to light up the rocket. At *t*_3_, the kerosene injected into strut-jet is turned off, and at *t*_4_, the oxygen injected into the rocket is turned off. The period of *t*_2_-*t*_3_ is to test whether the second combustion can be ignited by the fuel rich rocket plume while the period of *t*_3_-*t*_4_ is to test how the result will be when the rocket exhaust is pure oxygen.

**Figure 4:**
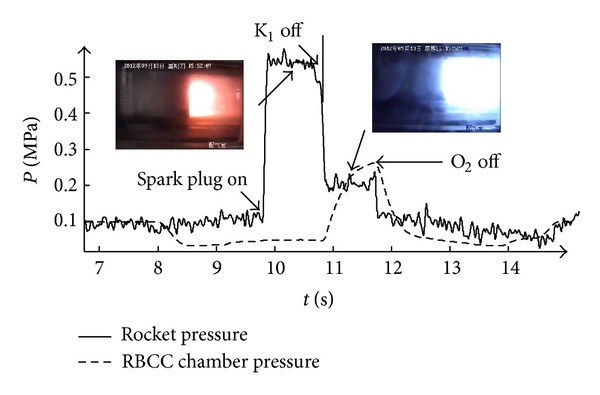
Pressure history of the strut-jet rocket base chamber and the RBCC combustor.

**Figure 5:**
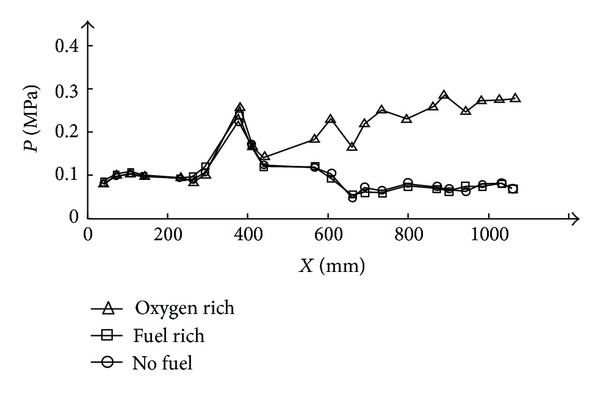
Pressure distribution under fuel/oxygen rich exhaust.

Results are shown by two pictures: Figure 4 shows the pressure change history along the time during the experiment. This figure contains the data of two pressure transducers: the dotted line is the 19th transducer, this pressure is mounted at the third part at *X* = 910 mm, this transducer is selected to detect whether the second combustion is founded, and the solid line is the pressure of the strut-jet rocket chamber, which is used to detect the working status of the rocket.  Figure 5 is the pressure distribution at typical time point. This picture is to contrast the pressure distribution in the RBCC combustor at the two different rocket plume conditions.

As shown in Figure 4, at about *t* = 9.5 s, the air heater has built up a steady flow; at this time, the pressure distribution along the combustor is shown in Figure 5 marked as “no fuel” state. The pressure variations indicate the effects of shock and expansion waves generated by the strut. The first shock generated by the strut leading edge interacts with the wall boundary layer, causing pressure rise of the 8th and 9th pressure sensors at about *X* = 370 mm. When kerosene and oxygen are injected into the combustor, there is no self-ignition although the total temperature of the incoming air flow reaches 1516 K. When the spark plug is turned on, the rocket is lit immediately. The pressure of the rocket chamber has reached about 0.55 MPa. Flame picture is also taken from the side window and the rocket flame is yellow; this is typical for fuel rich hydrocarbon due to the carbon particles. However the second combustion is not ignited and this can be reflected by dot line in Figure 4 and the pressure distribution in Figure 5. From Figure 4, the pressure used to detect the second combustion has not been affected during this period and the pressure distribution along the combustor has also shown that there is no remarkable different change compared with the no fuel condition. This means that for this fuel rich condition, the rocket plume is not able to found the second combustion.

At *t* = 10.8 s, kerosene injected into the strut-jet is shut off, only leaving the oxygen solenoid open. The pressure of the rocket chamber has rapidly reduced to about 0.2 MPa and this means that the reaction in the chamber has disappeared and the pressure rise of the rocket chamber is due to the oxygen injection. However the flame at the strut back did not disappear. Picture taken from the side window shows that the moment the kerosene is shut off, the color of the rocket plume has changed from yellow to light blue. Meanwhile, the pressure transducer used to detect the second combustion is affected, rises up to about 0.25 MPa, as shown in Figure 4.  Figure 5 shows that the pressure has an obvious rise downstream of the strut-jet. This means that the second combustion has been founded by this pure oxygen exhaust.

**Figure 6:**
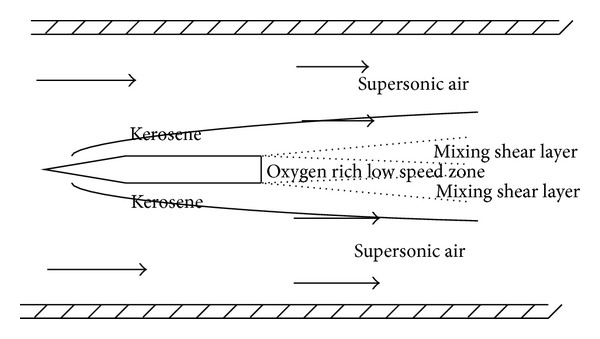
Flow structure at the rear part of the strut-jet.

As the result shows, in this way, the strut has been working as a flame stabilization torch. However, the torch is not a supersonic hot gas ejected form the rocket nozzle. It is more like an oxygen pilot flame. The flame stabilization mechanism of this local pilot flame can be explained by Figure 6.

Velocity of oxygen injected from the strut jet is about 40 m/s which is calculated based on the pressure and the entrance area which is much lower than the supersonic air flow. Because of the speed difference, there will be a shear layer between the high speed zone and the low speed zone and kerosene injected from upstream of the strut jet diffused into the shear layer begins to mix with the oxygen. Experiment result has shown that this local pilot flame can stabilize at the back of the strut-jet which has demonstrated that the reaction time is shorter than the residence time of the mixture. That is because the reaction speed of the kerosene and the pure oxygen is much faster compared with the air.

### 3.2. Local Flame and Second Combustion

**Figure 7:**
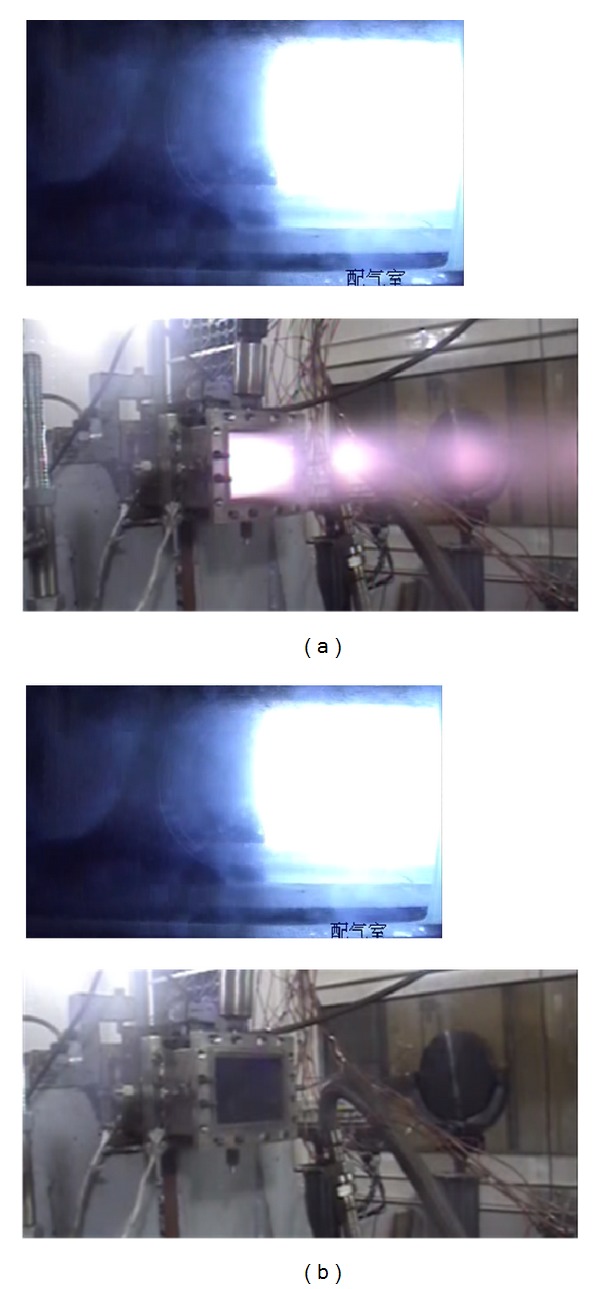
Local flame with/without second combustion ((a): with; (b): without).

However, the local fire does not mean the global combustion. The second combustion can only be trigged when the oxygen mass flow rate reached a certain degree.  Figure 7 has shown two combustion characteristics, one is the local fire with second combustion. At this situation there, is a remarkable pressure rise, and the flame of the second combustion can be seen with Mach disk at the exit of the RBCC combustor. The other one is local flame without second combustion. In this situation, though local fire can be seen at the strut-jet back from the side window, the pressure distribution is the same as the fuel off-state, and there is no flame at the exit. This situation occurs with a relative small oxygen mass flow rate.

**Figure 8:**
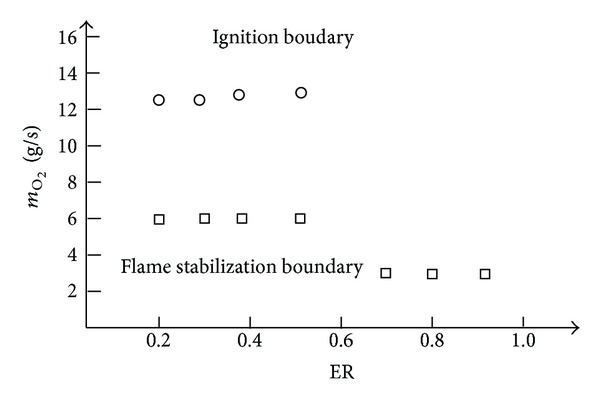
Oxygen mass flow rate need for ignition and flame stabilization at different ER.

Figure 8 gives the minimum oxygen mass flow rate required by successful ignition and flame stabilization of the second combustion. The horizontal axis is the ER, and the longitudinal axis is the oxygen mass flow rate. As the experiment result shows, compared with the oxygen mass flow rate needed for ignition, oxygen mass flow needed to sustain the flame stabilization is much smaller. Besides that, with the increase of ER, oxygen needed to sustain the second combustion is decreased.

**Figure 9:**
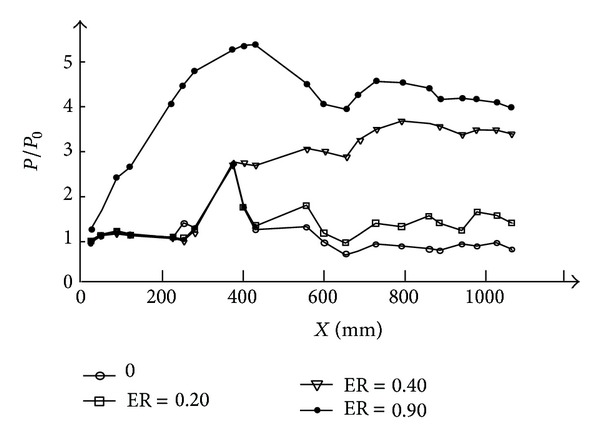
Pressure distribution under different ER.

**Figure 10:**
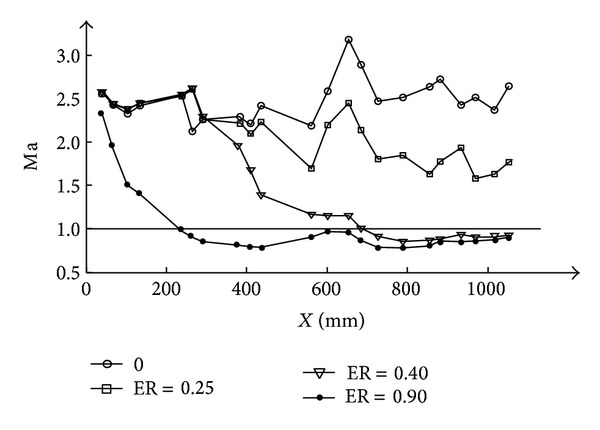
Mach number distribution under different ER.

This phenomenon can be explained by the flow character in the RBCC combustor.  Figure 9 shows the typical pressure distribution with the ER is 0.2, 0.4, and 0.9, respectively. All the wall pressure is normalized by the pressure of the first pressure transducer *P*_0_ where *X* = 30 mm. The corresponding Mach number distribution based on one dimensional calculation is shown in Figure 10.

Flow structure is much different before and after ignition of the RBCC combustor. Once the second structure is founded, there will be a series of shock trains in the flow path due to the heat release and the speed of the air flow slows down. Meanwhile, shock train itself can promote faster mixing and the boundary layer separation caused by interaction between the wall and shock train has formed additional low speed recirculation zone. All these flow structure changes can provide additional flame holding auxiliary effect [14–16].

The shock train intensity is mainly affected by the heat release of the second combustion. When the ER is low, for example, ER = 0.2, the pressure distribution curve along the combustor is jagged with an increase of mean pressure level by the end of the constant area. This pressure distribution characteristic indicates that combustion occurs in a supersonic flow with shocks, corresponding to a typical supersonic combustion mode. The calculated Mach number distribution also demonstrates that the flow is all supersonic in the whole combustor.

As ER increases, intensity of the shock train increases, the head of the shock moves upstream, and thermal throttling effect is getting stronger and stronger. At ER = 0.40, thermal choking occurs. As shown in Figure 10, the mean Mach number of the RBCC combustors is subsonic after *X* = 690 mm and the flow partly decelerated to subsonic. For ER = 0.90, the whole flow path after the strut-jet is subsonic. The combustion has entered into a typical subsonic combustion mode. These flow changes have shown that the intensity of the shock train is increasing with the increase of the ER, and the auxiliary combustion enhancement effects caused by the shock train are more and more obvious As a result, the requirement of the second combustion flame stabilization to the local flame intensity is reduced. The local flame intensity is mainly affected by the oxygen mass flow rate, so the oxygen needed to sustain the second combustion is decreased with the increase of ER.

## 4. Conclusion

In this paper, a strut-jet based RBCC combustor model is tested to investigate the flame stabilization characteristic with a small rocket mass flow rate. Mach number at the entrance of the combustor is 2.6 and liquid kerosene is used to fuel the strut rocket and the second combustion. Based on the experiment results, main conclusion can be drawn as follows.

1. The operating characteristic of the rocket is very important for the flame stabilization of the second combustion. With a small total mass flow rate, the fuel rich rocket plume cannot ignite the second combustion or even affect the entrained second air flow. But a pure oxygen exhaust can form a local pilot flame at the back of the strut-jet, and the second combustion can be triggered by this local flame.
2. Oxygen needed to ignite the second combustion and sustain the second combustion is different, and the requirement of the oxygen mass flow rate needed to sustain the second combustion is also different, decreasing as the increase of the ER. This phenomenon can be explained by the different intensity of the shock strain under different combustion modes. The existence of shock train has provided flame stabilization auxiliary. The intensity is mainly controlled by the ER of the second combustion, so the oxygen needed to sustain the second combustion is decreased with the increase of ER.
